# GnRH Agonist vs. hCG for Triggering of Ovulation – Differential Effects on Gene Expression in Human Granulosa Cells

**DOI:** 10.1371/journal.pone.0090359

**Published:** 2014-03-06

**Authors:** Jigal Haas, Libby Ophir, Eran Barzilay, Gil M. Yerushalmi, Yuval Yung, Alon Kedem, Ettie Maman, Ariel Hourvitz

**Affiliations:** Human Reproduction Lab and IVF Unit, Department of Obstetrics and Gynecology, Chaim Sheba Medical Center, Tel-Hashomer, Ramat Gan, Israel; Medical Faculty, Otto-von-Guericke University Magdeburg, Medical Faculty, Germany

## Abstract

**Objective:**

To investigate the mRNA expression of genes related to steroidogenesis and OHSS in granulosa cells (GCs) of patients triggered with GnRH agonist compared to patients triggered with hCG.

**Design:**

Mural GCs were obtained at the time of oocyte retrieval and gene expression was analyzed using quantitative real time RT-PCR.

**Settings:**

Single center, case control study.

**Patient(s):**

24 women who were treated with GnRH agonist or hCG for triggering of ovulation.

**Interventions:**

GC collection.

**Main Outcome Measure(s):**

The expression of genes related to steroidogenesis and OHSS in mural GCs

**Results:**

The fertilization rate was similar in the two groups. The mRNA expression of CYP19A1 (0.50 vs 1, arbitrary unit), CYP11A1 (0.6 vs. 1) and 3 beta hydroxysteroid-dehydrogenase (0.39 vs 1) was significantly lower in the GnRH group. The expression of VEGF (0.74 vs. 1) and inhibin β B (0.38 vs 1) was lower in the GnRH analog triggered group.

**Conclusion:**

Expression of genes related to steroidogenesis is lower at the time of oocyte retrieval in patients triggered with GnRH agonist. The decreased expression of VEGF and inhibin β B in the GnRH agonist group can explain the mechanism of early OHSS prevention.

## Introduction

Several studies have shown that the use of GnRH agonist for triggering of final oocyte maturation prevents the development of ovarian hyperstimulaion syndrome (OHSS), even in patients at high risk of developing OHSS [1]–[3]. In contrast to hCG, the GnRH agonist-induced surge resembles the natural mid cycle surge of gonadotropins and exposes follicles to both LH and FSH. However, after GnRH agonist triggering the mid cycle surge of gonadotropins is shorter in duration and amplitude compared with the natural cycle [4], [5]. The precise mechanism of OHSS prevention is not thoroughly understood. One of the hypotheses is that the shorter half-life of the endogenous LH surge induced by GnRH agonist, compared with the continuous high levels of hCG stimulating the LH receptor, induces a shorter and milder secretion of vasoactive substances such as vascular endothelial growth factor (VEGF) which is known among other proinflammatory cytokines to play a fundamental role in the pathophysiology of OHSS [6], [7].

Both inhibinA and inhibin B have been shown to be higher in patients developing OHSS. The level of inhibin B increase even before hCG administration and may serve as a predictor of OHSS, while inhibin A increases after developing OHSS and may be used to monitor the disease [8]

The LH surge plays a crucial role in the formation of the corpus luteum. A range of EGF-like ligands including AREG, EREG and β-cellulin function as LH target genes [9], and AREG is acting as a growth factor with an EGF-like motif that initiates the morphological and biochemical events triggered by the LH including cumulus expansion and oocyte maturation[10]. In addition, Follicle-Stimulating Hormone (FSH) and Luteinizing Hormone have a central role in follicle growth and maturation through interaction with their receptors, the Follicle Stimulating Hormone Receptor (FSHR) and Luteinizing Hormone Choriogonadotropin Receptor (LHCGR).

Although previous described that more mature oocytes were retrieved by the use of GnRH agonist for ovulation triggering [11], a poor clinical outcome with high early pregnancy loss rate was reported in this group of pregnancies [11]–[13]. These results can be explained by the luteal phase insufficiency caused by lysis of the corpus luteum which results in lower levels of progesterone in the group of women triggered with GnRh agonist. It is well established that progesterone formation depends on the activation of steroidogenesis genes such as STAR, which uptakes the cholesterol into the mitochondria, and CYP11A1, that converts cholesterol to pregnenolone. CYP19A1 converts androstendione to estradiol which is crucial for endometrium formation. Therefore, these steroidogenesis genes play a crucial role in the luteal phase and in early pregnancy stages.

The aim of this study was to investigate the mRNA expression of genes related to steroidogenesis and OHSS as well as to investigate other genes such as LH receptor (LHCGR), FSH receptor (FSHR), amphiregulin and epiregulin, at the early stage of 36 hours post triggering, in granulosa cells of patients triggered with GnRH agonist compared to patients triggered with hCG.

## Materials and Methods

The research was approved by the authors' institutional review board at Tel Hashomer hospital, IRB number 8707-11-SMC and written informed consent was obtained from each participating subject

### GnRH agonist or hCG triggering

The IVF patients included in the study were women <40 years of age undergoing IVF due to male factor infertility, tubal factor, or for the purpose of preimplantation genetic diagnosis (PGD, genetic tests of embryos prior implantation). Patients with infertility related to female pathology, such as poor ovarian response and endometriosis were excluded from the study. Intracytoplasmic sperm injection (ICSI) was performed only in cases of male factor infertility. All decisions regarding treatment protocol including mode of triggering were made according to physician preference. The treating physicians in the clinic were acting independently so that decisions regarding IVF protocol were taken impartially.

During the study period all patients who met the inclusion criteria sited above and were selected to receive agonist triggering by the treating physician according to his decision were recruited to the study. All 12 patients gave their consent and were recruited to the study. For the control group we recruited 12 consenting patients triggered with hCG while matching according to age, etiology of infertility, total dose of gonadotropins used for ovarian stimulation and length of treatment.

The granulosa cells (GCs) were obtained at the time of oocyte retrieval for IVF procedures. The women were treated using the antagonist protocol with GnRH antagonist (Cetrorelix; Merck Serono or Orgalutran; Schering-Plough). Ovarian stimulation with a daily SC dose of recombinant FSH (Gonal-F, Merck-Serono or Puregon Pen, Schering-Plough) and hMG (Menogon; Ferring) was started on the third day of the menstrual cycle. The amount of the initial dose depended on the age, body mass index (BMI), and treatment history. When three leading follicles reached 18 mm in diameter, the women received GnRH agonist (Decapeptyl 0.2 mg; Ferring) or hCG (Ovitrelle 250 mg; Merck Serono) for final oocyte maturation. Oocyte retrieval was performed by transvaginal ultrasound-guided needle aspiration, only follicles ≥16 mm were retrieved.

### Mural Granulosa Cell Purification

Mural GCs were collected from pooled follicular fluid [14], avoiding blood clots, and resuspended in phosphate-buffered solution (PBS). After allowing the cells to settle by gravity for a few minutes, the top medium was aspirated. This step was repeated two to three times until the medium was clear. The cells were centrifuged at 200 g for 5 minutes at room temperature, and the resulting pellets were resuspended in red blood cell (RBC) lysis buffer (Sigma-Aldrich). After a 15-minutes incubation period at 37°C, the cells were centrifuged at 200 g for 5 minutes at room temperature, and the resulting pellets were subjected to RNA purification.

The method we used for the isolation of the granulosa cells has been used in previous studies by us and by other authors [15]–[18].

### RNA Extraction and qRT-PCR

Total RNA was extracted from GCs by a Mini RNA Isolation I Kit (Zymo Research Corp.), according to the manufacturer's instructions. One microliter of RNA solution was used for reverse transcription with a high-capacity cDNA RT kit (Applied Biosystems) according to the manufacturer's instructions. A power SYBR Green PCR mix (Applied Biosystems) was used for the PCR step. Amplification and detection were performed using the StepOnePlus real-time PCR system (Applied Biosystems) with the following profile: 1 cycle at 95°C for 20 seconds, 40 cycles each at 95°C for 3 seconds, and 60°C for 30 seconds. One microgram of complementary DNA (cDNA) was used per reaction in a 10-mL reaction volume. All samples were run in duplicates. The β-actin RNA was chosen as a suitable normalization control gene. The same quantitative real-time PCR protocol was used for all the genes analyzed. Results are expressed as fold change with respect to the experimental control. For primers details, see Table S1.

In order to verify that the white blood cells (WBC) were removed, we measured the mRNA expression of CD45 (which identify WBCs) in the GCs and found negligible expression of CD 45 which demonstrated that the WBCs were removed from the samples.

### E2 measurement

The serum was analyzed for E2 concentration at the day of triggering using an E2 ELISA kit (Enzo Life Sciences Int, Farmingdale, NY) according to the manufacturer's instructions (Sensitivity 14.0 pg/ml (range 15.6–1,000 pg/ml)).

### Statistical analysis

Comparisons were performed using the unpaired two-tailed student's t-test assuming a normal distribution with unequal variances. A P≤0.05 was considered statistically significant.

## Results

There was no difference between the patients in the two groups in terms of age, duration of ovulation stimulation and the total dose of gonadotropins required for ovarian stimulation. In the group triggered with GnRH agonist, the average estradiol level at the day of ovulation triggering was significantly higher than in the group triggered with hCG (11853 pmol/L vs 8619 pmol/L, P<0.01). The number of eggs retrieved was similar in the two groups (13.75 vs 11.72 p = 0.35), the fertilization rate was similar in the two groups (Table 1). The women in the study group were at moderate risk of developing OHSS and in the control group, the risk for OHSS was lower demonstrated by lower levels of estrogen at the day of triggering.

**Table 1:** Characteristics of the patients and IVF procedures included in this study.

	Triggering with GnRH agonist			Triggering with Hcg			p value
n	12			12			NS
Age (mean)	31.8	±	3.3	31.7	±	4.8	Ns
E2 (mean)	11853	±	2075.5	8619	±	2757.3	0.006
oocytes retrieved	13.75	±	5.8	11.72	±	3.2	NS
duration of stimulation	11.58	±	1.8	11.2	±	1.7	NS
% fertilization	70	±	0.2	54	±	0.2	0.07

**Figure 1:**
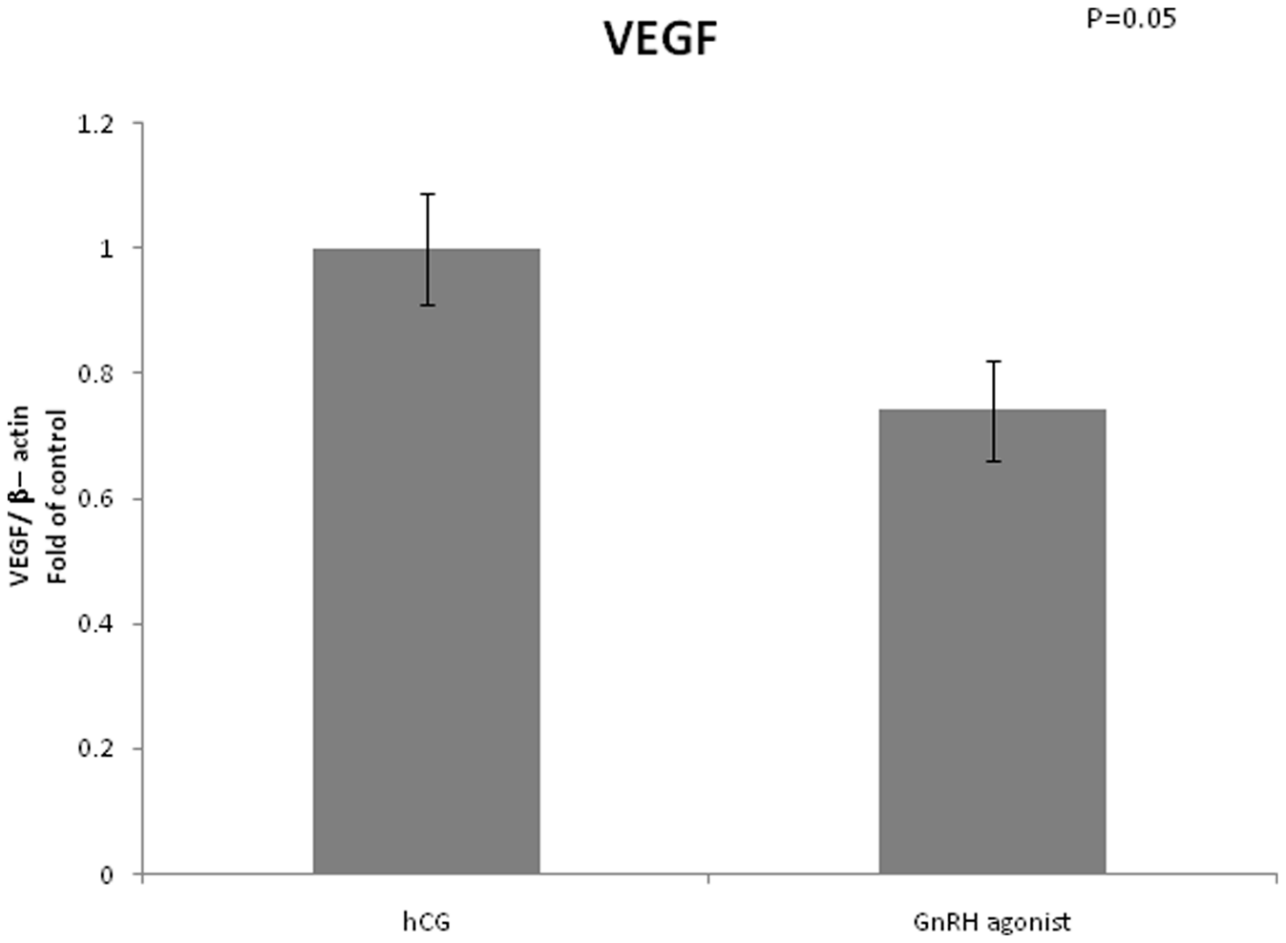
Expression of VEGF in mGCs obtained from IVF cycles separated according to ovulation triggering.

**Figure 2:**
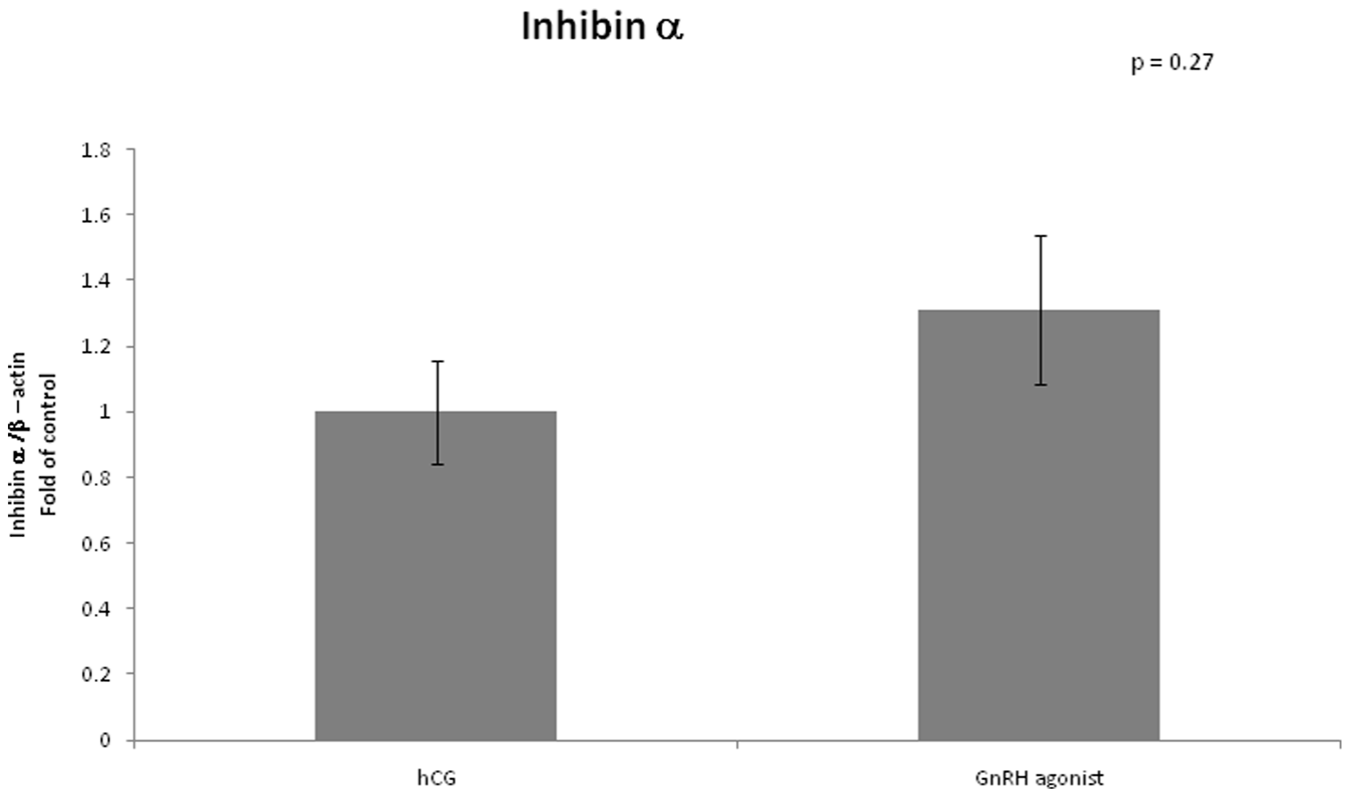
Expression of Inhibin α in mGCs obtained from IVF cycles separated according to ovulation triggering.

**Figure 3:**
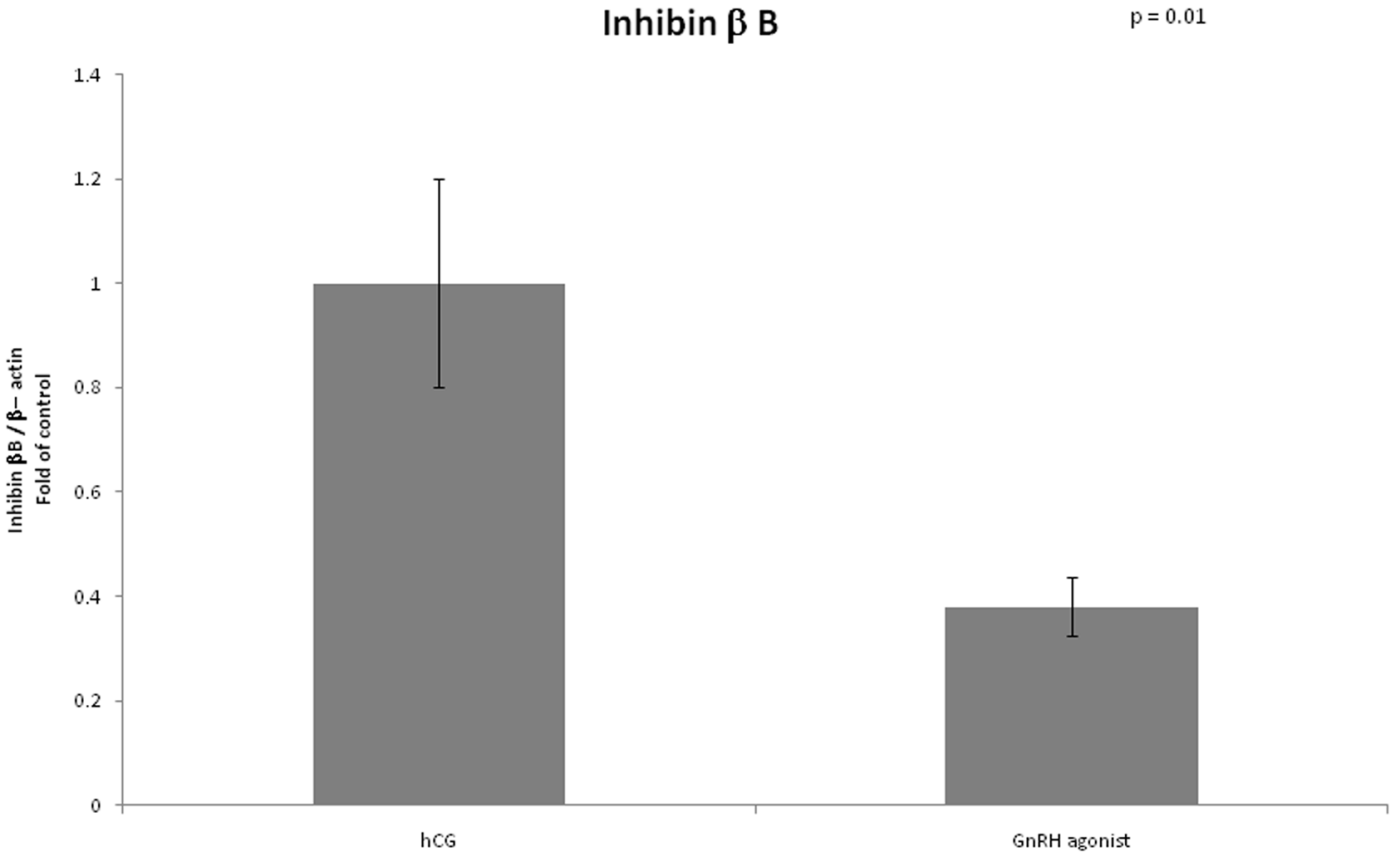
Expression of Inhibin β B in mGCs obtained from IVF cycles separated according to ovulation triggering.

VEGF mRNA expression was lower in GCs retrieved from women triggered with GnRH agonist compared to women triggered with hCG (0.74 vs 1, arbitrary unit, P = 0.05). Expression of inhibin β B was significantly lower in the GnRH agonist triggered group (0.38 vs 1, P = 0.01). We did not find any differences between the two groups regarding mRNA expression of inhibin α (Figures 1–3).

**Figure 4:**
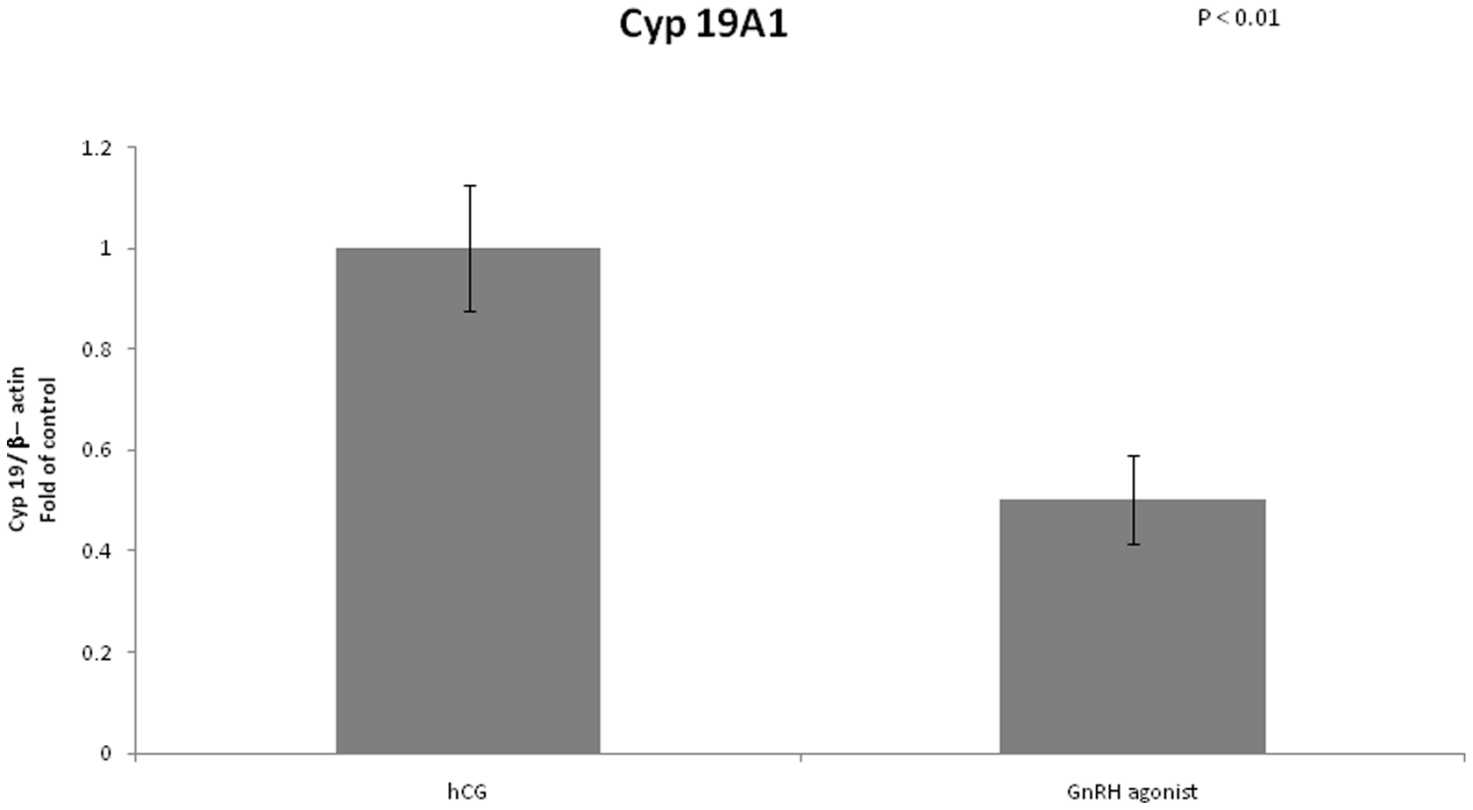
Expression of CYP19A1 in mGCs obtained from IVF cycles separated according to ovulation triggering.

**Figure 5:**
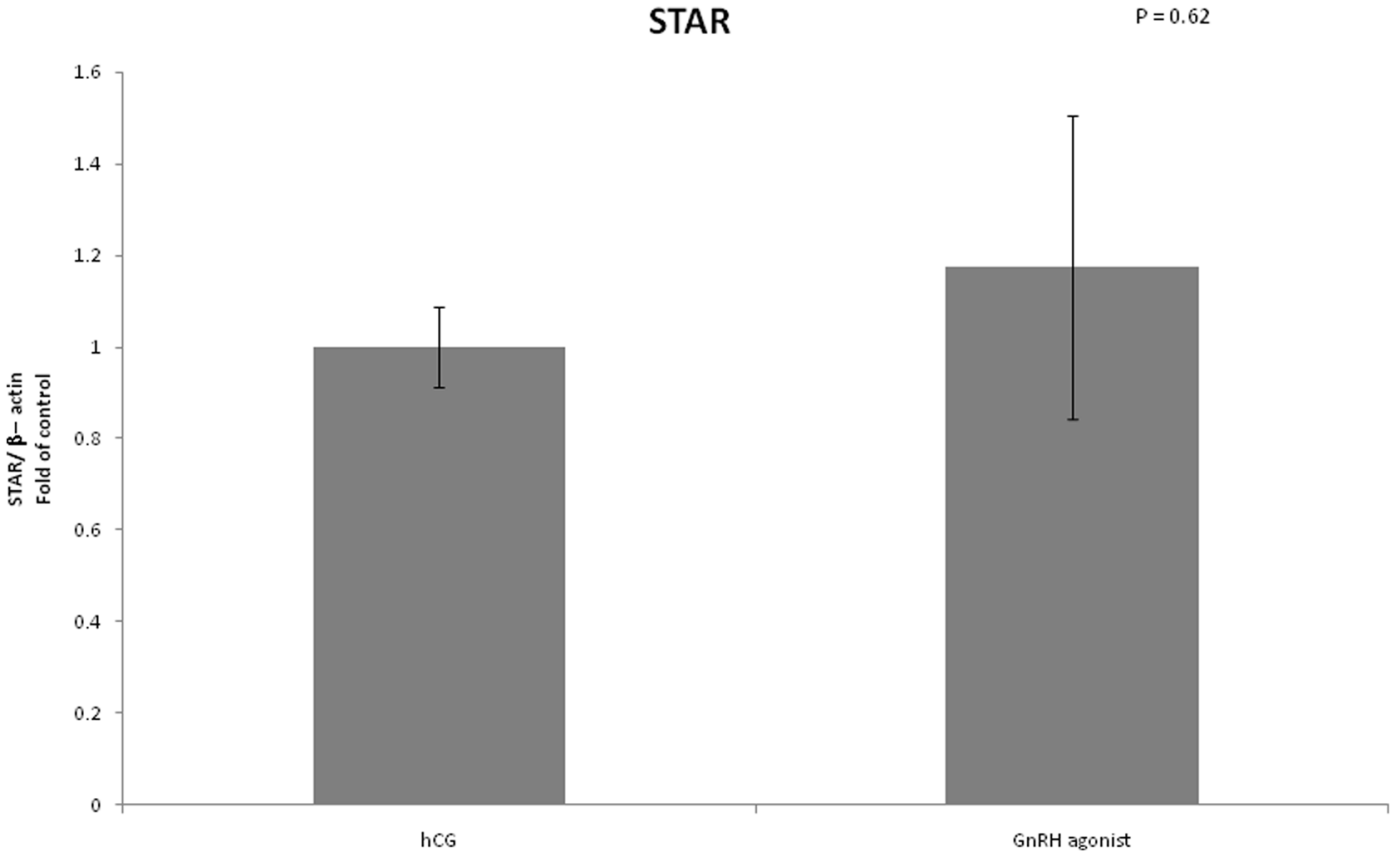
Expression of STAR in mGCs obtained from IVF cycles separated according to ovulation triggering.

**Figure 6:**
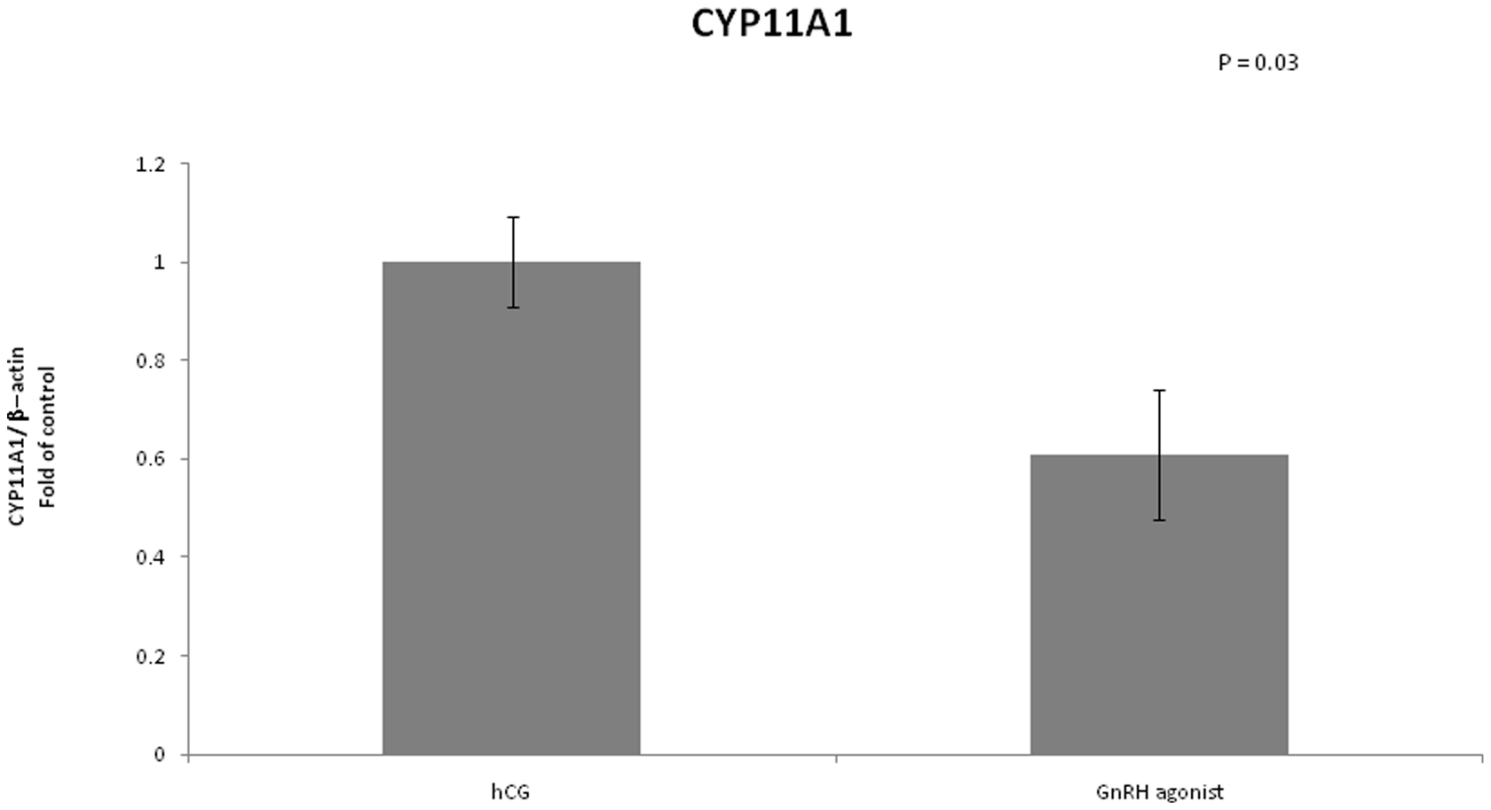
Expression of CYP11A1 in mGCs obtained from IVF cycles separated according to ovulation triggering.

**Figure 7:**
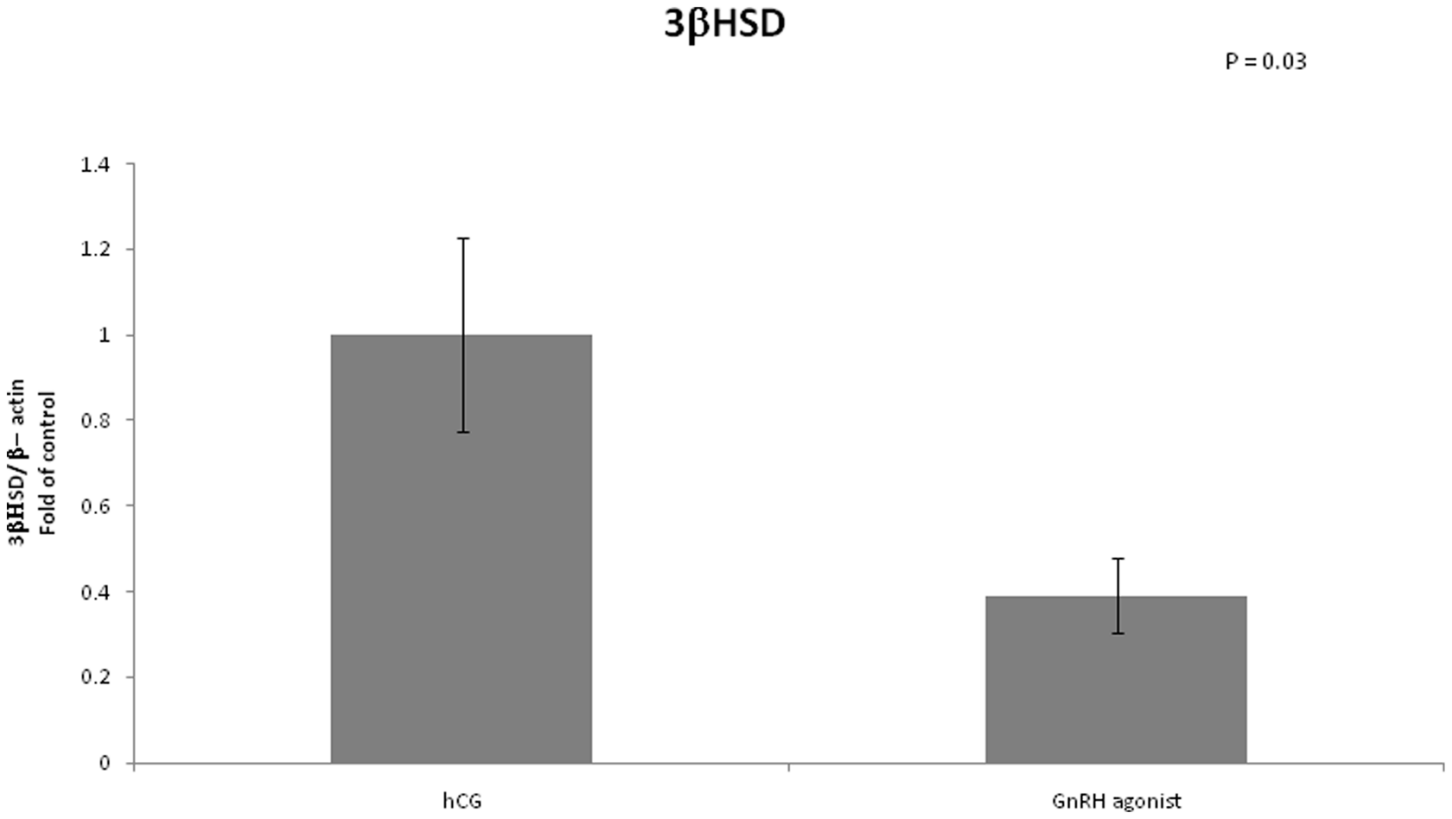
Expression of 3βHSD in mGCs obtained from IVF cycles separated according to ovulation triggering.

We examined the mRNA expression of different enzymes involved in steroidogenesis (Figures 4–7). The mRNA expression of CYP19A1 (0.50 vs 1, P<0.01), CYP11A1 (0.6 vs 1, P = 0.02) and 3 beta-hydroxysteroid-dehydrogenase (0.39 vs 1, P = 0.03) was significantly lower in the GnRH agonist group compared to the hCG group. Expression of STAR was similar in the two groups.

**Figure 8:**
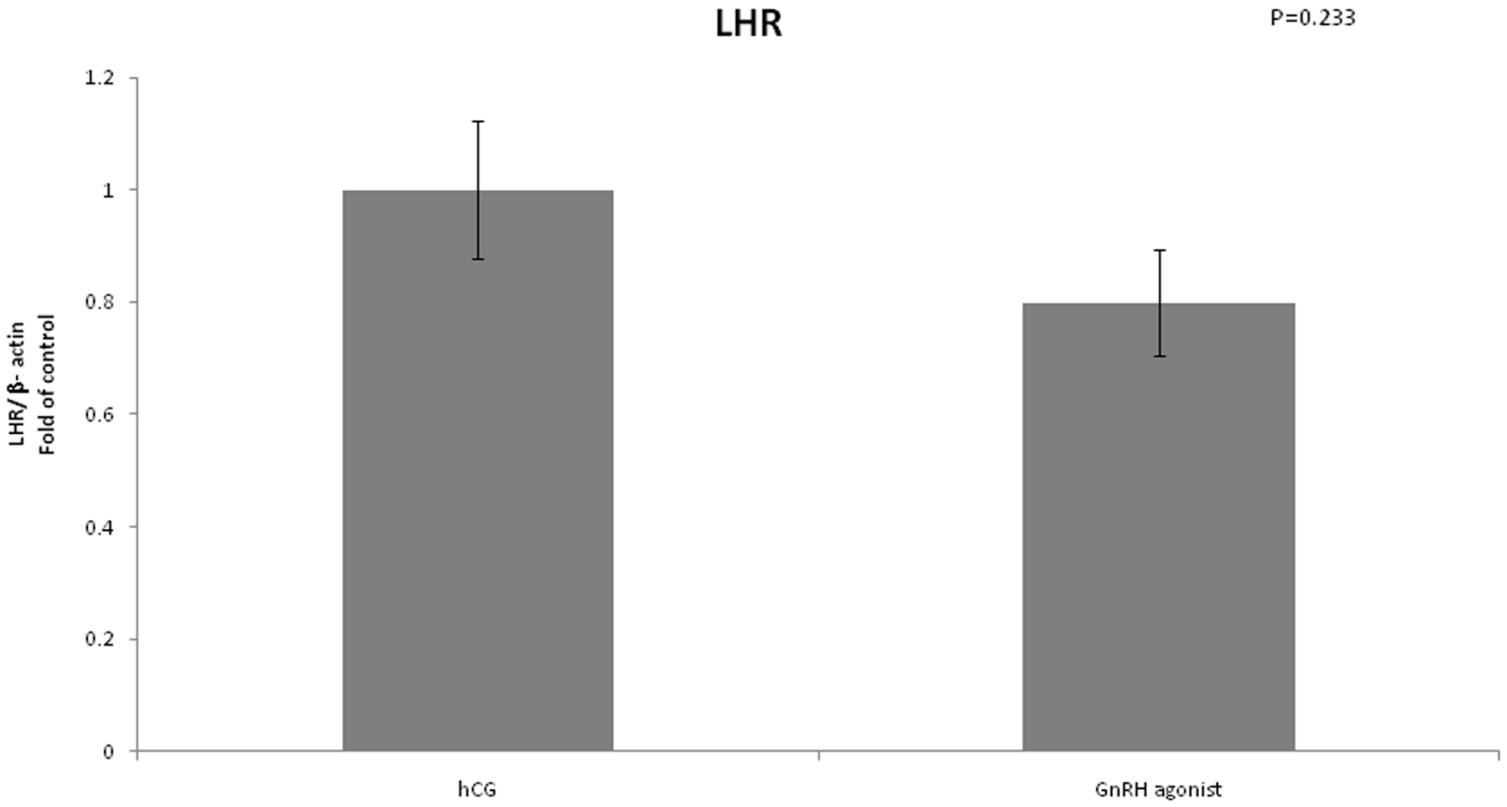
Expression of LHCGR in mGCs obtained from IVF cycles separated according to ovulation triggering.

**Figure 9:**
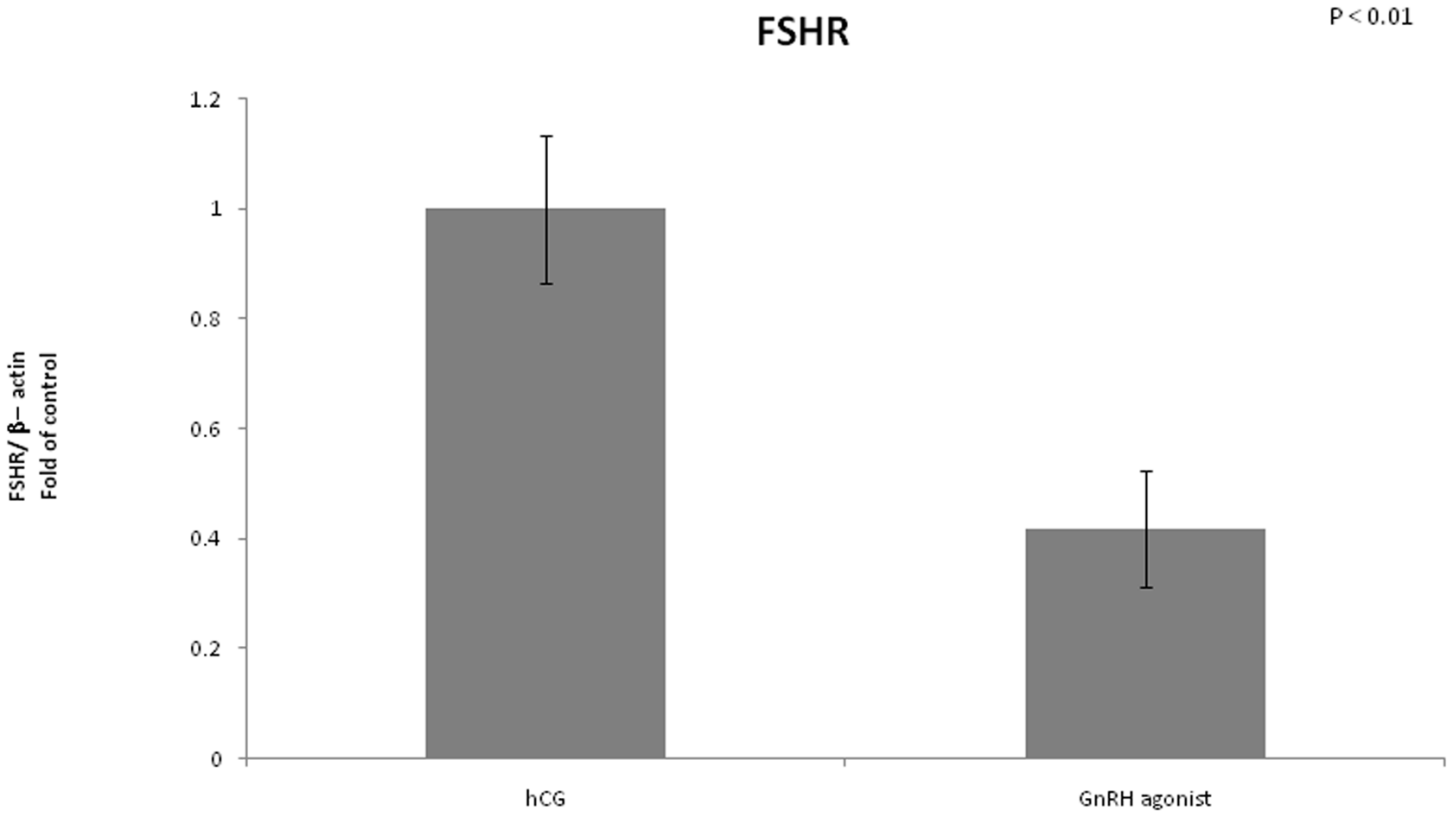
Expression of FSHR in mGCs obtained from IVF cycles separated according to ovulation triggering.

**Figure 10:**
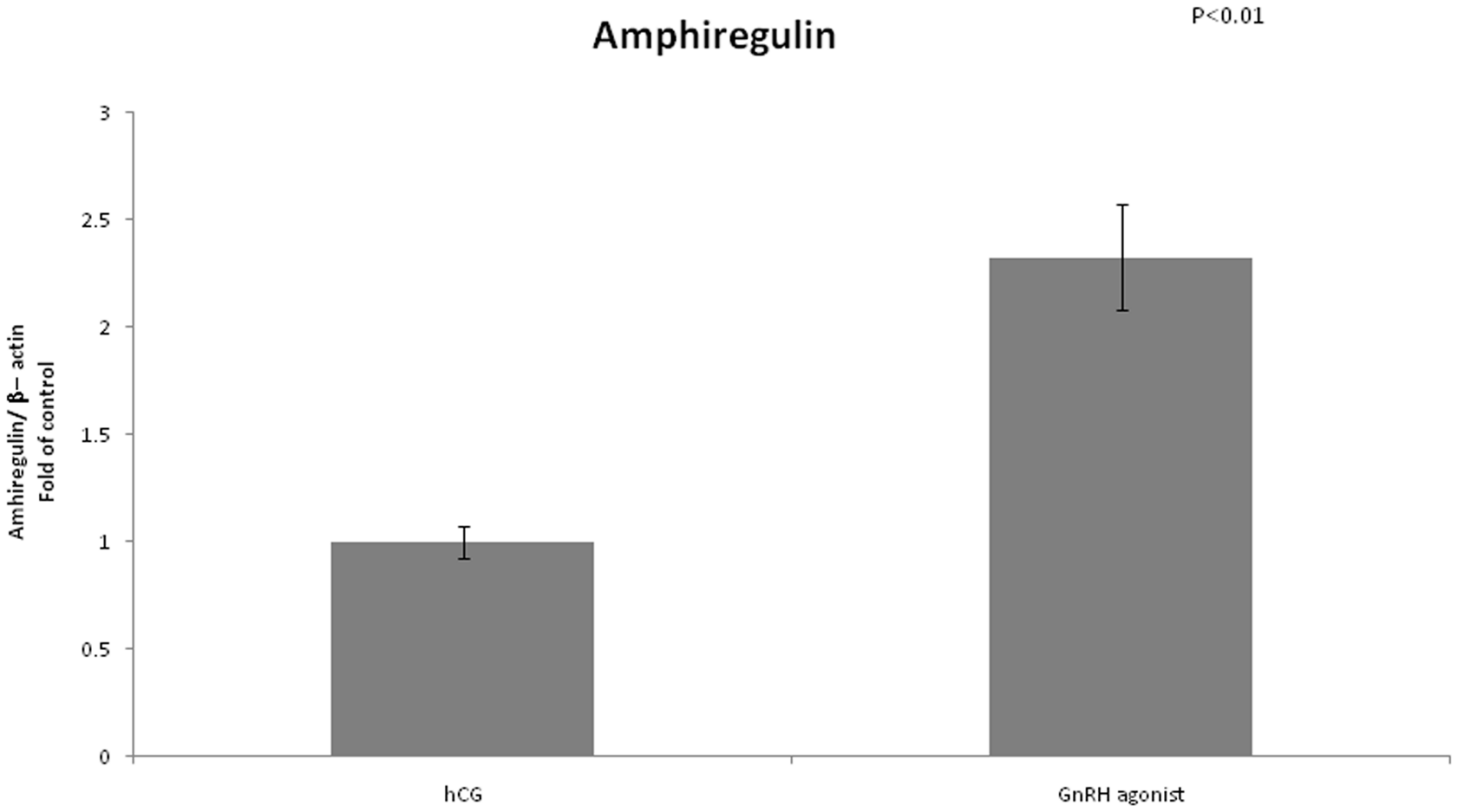
Expression of amphiregulin in mGCs obtained from IVF cycles separated according to ovulation triggering.

**Figure 11:**
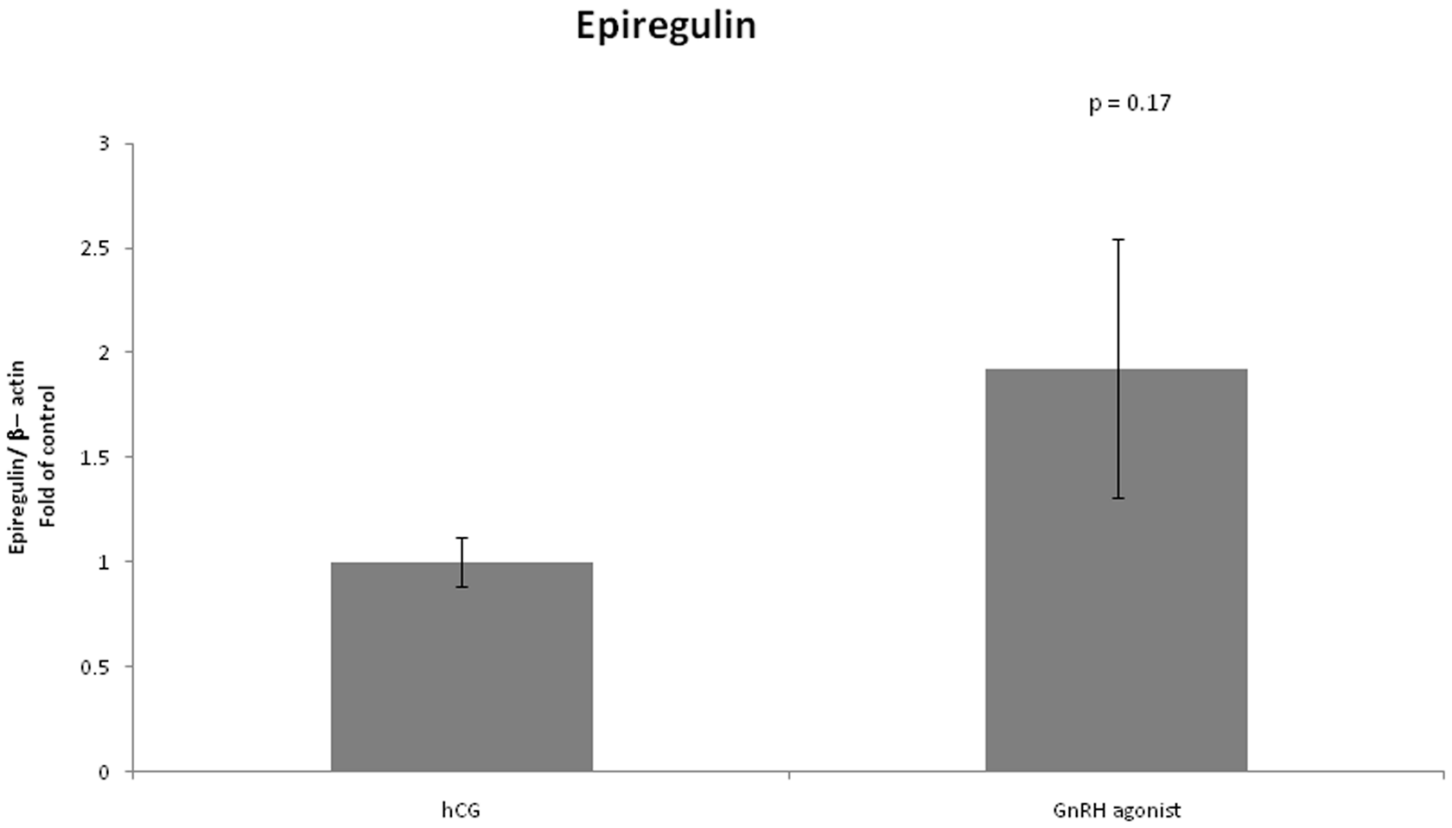
Expression of epiregulin in mGCs obtained from IVF cycles separated according to ovulation triggering.

We found no difference in expression of LHCGR between the two groups (Figure 8). FSHR mRNA expression was significantly lower in the GnRH triggered group compared to the hCG triggered group (0.4 vs 1, P<0.01) (Figure 9). Amphiregulin expression was significantly higher in the GnRH triggered group compared to the hCG triggered group (2.32 vs 1, P<0.01) (Figure 10). Despite the significant difference in amphiregulin expression in mural GCs, the level of amphiregulin in the follicular fluid was similar in the two groups. Epiregulin expression was higher in the GnRH triggered group compared to the hCG triggered group albeit with no statistical significance (1.92 vs 1, P = 0.17) (Figure 11).

To determine whether a link exists between the levels of estradiol and the mRNA expression of the different genes (amphiregulin, epiregulin, VEGF, LHCGR, FSHR) we divided the patients according to the serum estradiol levels on the day of triggering and according to the way of triggering. The patients with the highest (n = 4) and with the lowest(n = 4) levels of estradiol, in the group triggered with hCG were divided to two groups. One group (n = 4) consisted of patients with lower levels of estradiol (4400–5960 pmol/L) while the other group (n = 4) consisted of patients with higher levels of estradiol (11000–12050 pmol/L) on the day of triggering. We did not find any differences of expression between the two groups regarding all the genes that were evaluated. Patients triggered with GnRH agonist were divided to two groups at the same way. One group (n = 4) consisted of patients with lower levels of estradiol (8410–10700 pmol/L) while the other group (n = 4) consisted of patients with higher levels of estradiol (13810–15970 pmol/L) on the day of triggering. In this group also, we did not find any differences of expression between the two groups regarding all the genes that were evaluated.

## Discussion

To the best of our knowledge, this is the first study to systematically explore possible differences in mRNA expression of enzymes and receptors in granulosa cells of patients triggered with GnRH agonist compared to patients triggered with hCG.

This study demonstrates that the mode of ovulation triggering significantly affects mRNA expression of proteins that have been suggested to participate in the development of OHSS, such as VEGF and inhibin beta, as well as expression of important enzymes in the process of steroidogenesis, such as CYP19A1, CYP11 and 3 beta-hydroxysteroid-dehydrogenase, and expression of FSHR and amphiregulin.

### Prevention of OHSS

All studies published to date have shown that the use of GnRH agonist for triggering of ovulation prevents the development of ovarian hyperstimulaion syndrome even in patients which are at high risk of developing OHSS. The precise mechanism for preventing OHSS is not thoroughly understood.

Early studies have shown that VEGF might be responsible for the increase in vascular permeability and ascites development in women developing OHSS [7], [19]. Serum VEGF have been reported to be significantly higher in patients who developed severe OHSS than in patients at risk for OHSS who did not develop the syndrome [20], while other studies [21], [22] did not demonstrate elevated levels of VEGF in patients developing OHSS.

Previous studies comparing blood serum VEGF levels in women triggered with GnRH agonist to women triggered with hCG did not find any differences between the two groups [23], [24].

Few studies measured follicular fluid levels of VEGF in women triggered with GnRH agonist compared to women triggered with hCG. Some of the researchers found lower levels of VEGF in the group of women triggered with GnRH agonist [24], [25] while others did not find a difference between the two groups [26].

In our study we measured mRNA expression of VEGF and found it to be lower with borderline significance in the group who received GnRH agonist for final triggering. Our findings correlates with previous findings of Cerillo et al[25], that demonstrated significantly lower levels of VEGF mRNA expression in granulose cells obtained from women triggered with GnRH agonist compared with women triggered with hCG.

The lower levels of VEGF, which is known, among other proinflammatory cytokines, to play a fundamental role in the pathophysiology of OHSS, may explain why OHSS is prevented among women triggered with GnRH agonist. The lower VEGF mRNA expression may reflect the short LH signaling in women triggered with GnRH agonist.

Innhibin A consists of the alpha subunit and the A type of the beta unit, while inhibin B consists of the alpha subunit and the B type of the beta unit. The amino acid sequences of the beta subunits show 70% homology. The level of both inhibins was shown to be higher in patients developing OHSS. The level of inhibin B increase even before hCG administration and may serve as a predictor of OHSS, while inhibin A increases after developing OHSS and may be used to monitor the disease [8].

In our study we investigated mRNA expression of inhibin alpha, which was shown previously to be down regulated in OHSS in a rat model [27], and mRNA expression of inhibin beta B which expresses the presence of inhibin B.

As expected, we found the expression of inhibin β B to be significantly lower in the GnRH analog triggered group (0.38 vs 1, P = 0.01). The lower levels of inhibin β B among women triggered with GnRH agonist may play a role in prevention of OHSS.

### Lower steroidogenesis

The LH surge plays a crucial role in the formation of the corpus luteum. As mentioned before after triggering with GnRH agonist, there is a shorter duration of the LH surge compared to triggering with hCG, leading to a reduced LH support for the developing corpus luteum, which may cause early luteolysis. It has been suggested that luteolysis results in significantly lower levels of estradiol and progesterone after GnRH agonist triggering compared to triggering with hCG [23], [28], [29]. In our study we wanted to investigate how early the impairment of the steroidogenesis begins. We found differences in expression between the two groups as early as 36 hours after ovulation triggering, at the time of ovum pickup. Expression of CYP19A1, CYP11A1 and 3 beta-hydroxysteroid-dehydrogenase was significantly lower in the GnRH group compared to the hCG group. Engmann et al [30], showed that the granulose cells after triggering with GnRH agonist are still viable on the day of retrieval and that the rate of apoptosis of granulosa cells was comparable between the two groups. It is possible that at this early stage (Oocyte Pick Up), even though there are no overt signs of apoptosis, a cascade of cellular events that will eventually culminate in apoptosis has already begun. We propose that the different gene expression we found at this early stage might be one of the first signs of apoptosis.

Despite the apparent early lower levels of steroidogenesis, it is well known that rescue of the corpus luteum can be achieved by injecting a small dose of hCG at the day of the ovum pickup [28], [31]. The fact that corpus luteum rescue is possible may imply that the early luteal dysfunction in the GnRH agonist triggered group as demonstrated in this study is reversible.

### LH and FSH receptors

In our study, LHCGR expression was similar in both the GnRH triggered group and the hCG triggered group, but FSHR expression was significantly lower in the GnRH triggered group compared to the hCG triggered group (0.4 vs 1, P<0.01). We suggest that the supraphysiologic levels of FSH which occurs after triggering with GnRH agonist may results in negative feedback which culminates in lower levels of FSH receptors.

### Amphiregulin

Amphiregulin and epiregulin are ligands of the epidermal growth factor receptor, released from mural GCs. LH stimulation of GCs induces up regulation of amphiregulin and epiregulin. It was previously suggested that they mediate the LH signal and partially take a part in the process of cumulus expansion and oocyte maturation [9]. The concentration of amphiregulin in the follicular fluid has been shown to correlate inversely with the fertilization rate whereas little significant association was observed between the level of amphiregulin and embryo quality [32]. In contrast, Ben Ami *et al.* [33] demonstrated that enrichment of maturation medium with amphiregulin and epiregulin significantly improved the maturation rate of human Germinal Vesicle oocytes *in vitro*. Humaidan *et al.* measured the levels of amphiregulin in the follicular fluid after triggering of final oocyte maturation with either GnRH agonist or hCG. Significantly lower levels of amphiregulin were found in FF from the GnRH agonist group as compared to the hCG group. Amphiregulin concentration in FF was negatively correlated with the fertilization rate. Moreover, a trend for an inverse correlation was found for amphiregulin and embryo quality[26].

In our study amphiregulin expression was significantly higher in GCs retrieved from women who were triggered with GnRH agonist compared with women triggered with hCG (2.32 vs 1, P<0.01). We did not find any difference in the levels of amphiregulin in the FFs. Combining the two groups of women, regardless of the route of triggering, we found a positive correlation between levels of mRNA expression of amphiregulin and fertilization rate. The significantly higher mRNA expression of amphiregulin in the GnRH agonist triggered group is in a distinct contrast to previously published data regarding the relations between amphiregulin, fertilization rate, embryo quality and mode of ovulation triggering. However, this discrepancy may be explained by the fact that in our study we examined mRNA expression while other studies examined amphiregulin levels in the FF. It is well known that downregulation of EGF receptor signaling occurs through trafficking of the receptor-ligand complex to lysosomes, culminating in proteolytic destruction of both the receptor and ligand [34], [35]. Receptor activation may therefore induce uptake and degradation of amphiregulin, which, in a confined space such as in the follicle, may induce a significant decrease in amphiregulin levels. Thus, our findings do not contradict previous published data since increased production of amphiregulin is not directly linked to increased levels of the protein in the FF. Based on our findings regarding expression of amphiregulin and the findings of Ben Ami *et al*. regarding addition of amphiregulin to maturation medium, we suggest that amphiregulin is important to oocyte maturation and that the contrasting data regarding amphiregulin levels in FF may be due to ligand uptake and degradation by cumulus cells.

Like all other non randomized studies there might be a selection bias in our study. Therefore we matched the hCG triggered group to the GnRH agonist triggered group according to age, etiology of infertility, total dose of gonadotropins used for ovarian stimulation and length of treatment. In the results section we described the basic parameters of the women in the two groups and as shown except of the E2 levels, all the other parameters were similar proving the efficiency of the matching technique. To deal with the higher E2 levels in the GnRH agonist group we divided the patients according to the serum E2 levels on the day of triggering and compared the expression profile of the genes. We did not find any differences of expression between the two groups regarding all the genes that were evaluated.

However, we can't rule out completely any selection bias and therefore farther study with patient randomization should be performed to strengthen our results.

In conclusion, our findings suggest that the decreased expression of VEGF and inhibin β B in the GnRH agonist group can explain the mechanism of early OHSS prevention in these women. The expression of enzymes which take part in steroidogenesis of estrogen and progesterone is lower at the time of oocyte retrieval in patients triggered with GnRH agonist. Taken together, we have shown that gene expression in granulosa cells is strongly linked to the method of ovulation triggering. The fact that gene expression pattern is profoundly different between hCG and GnRH agonist triggered women raises the possibility that granulosa function may also be linked to ovulation triggering method. The cellular and clinical effects of these differences of expression as well as the possible effect on oocyte function and quality remains to be further studied.

## Supporting Information

Table S1 **Real time polymerase chain reaction primers sequence.**
(DOCX)
