# Supplementary material for: GnRH Agonist vs. hCG for Triggering of Ovulation – Differential Effects on Gene Expression in Human Granulosa Cells
Source: PLoS One. 2014 Mar 6;9(3):e90359. doi: 10.1371/journal.pone.0090359 (PMC3946044; doi:10.1371/journal.pone.0090359)
Supplement: Table S1 — Real time polymerase chain reaction primers sequence. (DOCX) [file pone.0090359.s001.docx]

Supplemental Table 1: Real time polymerase chain reaction primers sequence

| Gene Name | Primers sequence | length | Accesion # |
| --- | --- | --- | --- |
| LHCGR | sense 5'- TGGAGAAGATGCACAATGGA  antisense 5'- GGCAATTAGCCTCTGAATGG | 122 | NM_000233 |
| FSHR | sense 5'- GGTGCATTTTCAGGATTTGG  antisense 5'- TTGGGAAGGTTGGAGAACAC | 98 | NM_000145.2 |
| AREG | sense 5'- AGCCGACTATGACTACTCAG  antisense 5'-CTTAACTACCTGTTCAACTCTGAC | 94 | NM_001657 |
| EREG | sense 5'- ACAGGCAGTCCTCAGTACAACTGTG  antisense 5'- TGACACTTGAGCCACACGTGGAT | 178 | NM_005228.3 |
| CYP11A1 | sense 5'- TGGGTCGCCTATCACCAGTAT  antisense 5'- CCACCCGGTCTTTCTTCCA | 82 | NM_000781.2 |
| Cyp19A1 | sense 5'- TGCAAAGCACCCTAATGTTG  antisense 5'- TGGTACCGCATGCTCTCATA | 135 | NM_000103.3 |
| STAR | sense 5'- ATTCAAGCTGTGCGCTGGGAGC  antisense 5'- TGGCCATCACAGCCTGTTGCC | 77 | NM_000349.2 |
| 3BHSD | sense 5'- TCATCCGCCTCTTGGTGAAGG  antisense 5'- AGCTTGGTCTTGTTCTGGAGTT | 112 | NM_000862.2 |
| VEGF | sense 5'- TGGGCCTTGCTCAGAGCGGA  antisense 5'- GCTCACCGCCTCGGCTTGTC | 150 | NM_001025366.2 |
| Inhibin α | sense 5'- GGAGGGCAGAAATGAATGAA  antisense 5'- CCTTGGAAATCTCGAAGTGC | 141 | NM_002191.3 |
| Inhibin β B | sense 5'- GCGCGTTTCCGAAATCATCA  antisense 5'- TTCTGGTTGCCTTCGTTGGA | 96 | NM_002193.2 |
| CD45 | sense 5'- CATATGACTATAACAGAGTGCC  antisense 5'- ATGTATTTGCTTGGTTCCTC |  | NM_002838 |
